# JA Signaling Inhibitor JAZ Is Involved in Regulation of AM Symbiosis with Cassava, Including Symbiosis Establishment and Cassava Growth

**DOI:** 10.3390/jof11080601

**Published:** 2025-08-19

**Authors:** Yu Gao, Siyuan Huang, Jingling Zhang, Lin Zhu, Baocan Zhan, Xiaohui Yu, Yinhua Chen

**Affiliations:** 1 State Key Laboratory of Tropical Crop Breeding, Sanya Institute of Breeding and Multiplication, Hainan University, Sanya 570025, China; 184394@hainanu.edu.cn (Y.G.); 18826074240@163.com (S.H.); zjling0128@163.com (J.Z.); 23220951310078@hainanu.edu.cn (L.Z.); mc668320@163.com (B.Z.); 2 School of Tropical Agriculture and Forestry, Hainan University, Haikou 570228, China

**Keywords:** cassava, arbuscular mycorrhizal fungi, symbiosis, jasmonic acid, JAZ

## Abstract

Mutualism between plants and arbuscular mycorrhizal fungi (AMF) is imperative for sustainable agricultural production. Jasmonic acid (JA) signal transduction has been demonstrated to play an important role in AMF symbiosis with the host. In this study, SC9 cassava was selected as the research object to investigate the effect of the jasmonic acid signaling pathway on symbiosis establishment and cassava growth in AMF and cassava symbiosis. It was first found that the symbiosis of cassava and mycorrhizal fungi could increase the biomass of both the aboveground and belowground parts of cassava. Secondly, JA content increased significantly in the early stage of AMF inoculation and auxin content increased significantly in the late stage of AMF inoculation, suggesting that JA signal transduction played an important role in the symbiosis between cassava and mycorrhizal fungi. Transcriptome data were used to analyze the expression differences of genes related to JA synthesis and signal transduction in cassava. The *MeJAZ* gene positively responded to symbiosis between cassava and mycorrhizal fungi. The analysis of MeJAZ gene family expression and its promoter supported this result. Spraying different concentrations of MeJA on leaves could affect the colonization rate and root biomass of cassava, indicating that JA was an active regulator of mycorrhizal formation. PPI prediction and qPCR analysis suggested that the *MeJAZ7* gene might be a key transcriptional regulator responding to jasmonic acid signals and regulating mycorrhizal influence on cassava growth and development.

## 1. Introduction

*Manihot esculenta* Crantz is an important energy source and security crop that supports the livelihoods of more than 800 million people worldwide [1,2], especially low-income groups in tropical and subtropical regions. Its root tuber is rich in starch and has the characteristics of maintaining a relatively high yield under abiotic stresses such as poor soil and drought [3,4]. However, cassava production faces multiple challenges, such as pest threats, low nutrient use efficiency, and intensified climate change, which seriously restrict its stable and sustainable production [5,6,7,8]. Against this background, the inherent biological potential of cassava interacting with beneficial microorganisms, especially its ancient symbiotic relationship with arbuscular mycorrhizal fungi (AMF) [9], is becoming a key avenue for improving cassava stress resistance and productivity.

Arbuscular mycorrhizal (AM) symbiosis is among the most widespread and important plant–microorganism mutualisms in terrestrial ecosystems [10,11]. AMF significantly expand the absorption range of plant roots through their huge hyphae network, efficiently assisting the host in obtaining mineral nutrients such as phosphorus, nitrogen, and zinc, which are poorly mobile in soil and water. In return, the plant provides carbon sources for photosynthesis to fungi [12,13,14,15]. In cassava, AM symbiosis not only promoted growth, but also increased nutrient (especially phosphorus) uptake efficiency [16,17], and has been shown to enhance tolerance to drought [18,19], salt stress [20,21,22], and soilborne diseases such as bacterial wilt caused by Xanthomonas [23,24,25]. Therefore, analyzing the molecular regulatory network of cassava–AM symbiosis will provide a theoretical basis for maximizing the use of this symbiosis system to enhance cassava stress resistance and productivity.

Although AM symbiosis exists widely in nature and is essential for cassava growth, there are significant differences in symbiosis efficiency among cassava varieties and under different environmental conditions, and its internal regulatory mechanism has not been fully clarified. Recent studies on rice have further highlighted that AMF can enhance plant growth and phosphorus use efficiency by activating specific regulatory genes and nutrient transport pathways [26]. Establishing and maintaining symbiotic relationships are highly programmed and complex processes involving elaborate molecular dialogue and signal integration between host plants and fungi [27,28,29]. Plant hormone signaling networks have recently been identified as central hubs for coordinating symbiotic development [30,31,32]. Jasmonic acid (JA) is a key defense hormone [33,34]. Its role in AM symbiosis presents complex and subtle “double-edged sword” characteristics: moderate JA signals may participate in the transmission of early recognition signals or promote the expression of symbiosis-related genes, while excessive or persistent JA signals may activate plant defense responses, thus inhibiting the development and function maintenance of mycorrhizal structures [35,36,37]. This precise balance regulation is essential for successfully establishing symbiotic relationship stability [38].

Jasmonate ZIM-domain (JAZ) protein, a core inhibitor of the JA signaling pathway, plays a central role in dynamic perception and response to changes in JA levels [34,39]. In the low JA state, JAZ protein binds to transcription factors such as MYC2 and suppresses the expression of downstream JA responsive genes [40]; when JA levels are elevated, JAZ protein is recognized and degraded by the SCFCOI1 E3 ubiquitin ligase complex, relieving the repression of transcription factors and activating the JA response [40]. Studies have shown that in model plants such as alfalfa and tomato, specific JAZ family members precisely regulate the expression network of symbiosis-related genes by integrating JA signals with other symbiosis-related hormonal signals (such as strigolide SLs and cytokinin CKs), thereby affecting mycorrhizal colonization [36,41]. However, in cassava, an important crop, whether and how members of the large JAZ gene family (MeJAZs) participate in, as well as which key members specifically regulate, the establishment and maintenance of cassava AM symbiosis remain mysteries, which seriously restricts the development of strategies for the targeted optimization of cassava symbiosis efficiency through molecular means.

The role of phytohormones in regulating interactions between plants and beneficial microorganisms has attracted much attention. JA, a typical defense signaling molecule, has also been found to be involved in coordinating signals in the symbiosis between plants and AMF [35,42]. In this study, we observed that following inoculation with AMF, the aboveground and underground biomass of cassava increased significantly. Additionally, the hormone levels in cassava exhibited dynamic changes, indicating that JA increased significantly in the early stage of symbiosis and that auxin accumulated gradually in the late stage, suggesting that JA may play a leading role in the early stage. Based on these findings, we hypothesized that the JA signaling pathway plays a key regulatory role in cassava–AMF symbiosis. Specifically, JAZ transcription inhibitors may act as signal response nodes to mediate cassava transcriptional responses to symbiotic stimuli, thereby regulating the establishment and maintenance of symbiosis.

To verify this hypothesis, this study used the cassava–AM symbiosis system as a model to analyze the expression characteristics and potential functions of the JAZ protein, a key factor in the JA signaling pathway. This was undertaken in order to reveal the mechanism of stress response and growth regulation in the early and stable stages of symbiosis, providing theoretical support for JA-mediated symbiosis regulation.

## 2. Materials and Methods

### 2.1. Plant Materials and Inoculation

Cassava variety: The cassava variety used in this study was South China No. 9 (SC9), collected from the Haidian Campus base of Hainan University and the cassava germplasm resource garden of Danzhou Campus of Hainan University.

Preparation of cassava seed stems: Cut the mature and lignified cassava stems into stem segments of approximately the same length and size, ensuring each segment has 3 to 5 effective bud points. Soak the segments in a multi-fungicide solution for 30 min to remove bacteria, fungi, and insect eggs from the stem. Plant the segments directly into pots containing a sterilized growing medium. Maintain a temperature of 28 °C, with 16 h of light daily, and 8 h of darkness. Apply the same amount of low-phosphorus Hoagland nutrient solution (the formulation is detailed in Table S1) every 3 days.

Expanded host plants: Corn and scallions, purchased from the general market.

The tested strain: *Rhizophagus irregularis* (Ri). This is a standard AMF strain widely used internationally. The AMF strain used in this study was isolated and identified by our laboratory from cassava rhizosphere soil collected in Luwang Village, Hongqiao Town, Pingding Township, Yujiang District, Yingtan City, Jiangxi Province. It was then propagated and cultured for use in this study.

Culture medium: River sand and vermiculite or zeolite (V:V = 4:1). After thorough cleaning, sterilize in a high-temperature and high-pressure sterilizer at 121 °C for 40 min twice.

### 2.2. Determination of Plant Vigor and Biochemical Indicators in Cassava Seedlings

(1) Cassava seedling treatment methods.

Mature lignified cassava stems of the same size and thickness were divided into the following two groups: the control group was not inoculated with AMF, and the treatment group was inoculated with AMF (*Rhizophagus irregularis*, Ri). They were planted vertically in flowerpots to ensure they were the same height above ground. Plant growth and hormone determination were carried out at two time points, the symbiosis establishment stage and stable stage, respectively, to compare the growth differences between inoculated and uninoculated treatments.

(2) Measurement of plant vigor in cassava seedlings.

Plant height: We measured the height from the base to the top of the stem with a ruler (accurate to 0.01 cm). At least three plants were selected from each group, each plant was measured 3 times, and the average value was taken.

Stem thickness: Measure stem diameter at a distance of about 1 cm from the growth point using vernier calipers (accurate to 0.01 mm) in the same manner as above. Above-ground fresh weight: cut off all stems and leaves, weigh them with an electronic analytical balance (accurate to 0.01 g), measure at least 3 plants in each group, measure 3 times for each plant, and take the average.

Belowground fresh weight: reduce watering 2–3 days before sampling, take out the root system completely, clean it, suck up the surface moisture, and weigh it (same as above).

(3) Determination of Biochemical Indicators in cassava seedling root systems.

The cassava root samples were collected at the symbiotic establishment and stable stages. Three biological replicates were set up for each treatment. Each replicate consisted of three samples, and the fresh weight of a single sample was not less than 2 g. The samples were frozen and stored at −80 °C. The endogenous hormone contents in the roots were measured by enzyme-linked immunosorbent assay (ELISA).

### 2.3. Staining of Arbuscular Mycorrhizal Fungi and Determination of Infection Rate

Cassava roots were placed in 50 mL centrifuge tubes and fully submerged in 10% (*w*/*v*) KOH solution. The samples were incubated in a 90 °C water bath for 5–10 min for tissue decolorization, followed by 3–5 rinses with ddH_2_O to remove residual KOH. The roots were then transferred to 1 mol/L HCl solution, incubated at room temperature for 15–30 min, rinsed again with ddH_2_O, and soaked in 1 × PBS for 15–30 min. The root samples were transferred to sterile 2 mL centrifuge tubes containing WGA-Alexa Fluor 488 staining solution (prepared at a 1000:1 ratio of PBS to dye) for staining. The roots were gently spread to ensure complete contact with the dye and incubated overnight at 4 °C in the dark.

After dyeing, the roots were dispersed in sterile water, selected root segments were placed on glass slides, and 10% glycerol was added to seal the slides. Typical mycorrhizal structures such as arbuscular branches, vesicles, and hyphae were observed under a fluorescence microscope. In total, 30 root segments were randomly selected from each sample for observation, 3 biological replicates were set, and the average of these replicates was used as the final infection level of the sample. The infection rate and infection intensity were calculated according to Trouvelot. The relevant statistical data were analyzed by MYCOCALC software (https://www2.dijon.inrae.fr/mychintec/Mycocalc-prg/download) (accessed on 30 April 2025).

### 2.4. RNA Extractions and Gene Expression Quantification

The extension factor 1-α (*EF-1α*) was selected as the standardized internal reference gene for expression data, and the genes of the MeJAZ gene family were selected for real-time fluorescence quantitative PCR. qRT-PCR-specific primers were designed for the selected genes using NCBI Primer-BLAST (https://www.ncbi.nlm.nih.gov/tools/primer-blast/index.cgi?LINK_LOC=BlastHome) (accessed on 5 March 2025) (Table S2). Reverse transcription was performed using the HiScript Ⅲ RT SuperMix for qPCR (+gDNA wiper) kit using total cassava RNA as the template. Semi-quantitative PCR detection was first conducted using the internal reference gene to test the abundance of cDNA. Based on the electrophoresis gel diagram after detection, it was determined whether we could continue to use the sample for the subsequent real-time fluorescence quantitative PCR reaction (Table S3).

The template was diluted according to the required quantity for quantification. Changes in the expression levels of differentially expressed genes were detected using the Servicebio”2xUniversal Blue SYBR Green qPCR Master Mix and the high-throughput fluorescence quantitative gene amplification instrument qTOWER384G (Analytikjena). We set up three technical replicates for each group of samples and placed them in a quantitative PCR instrument for amplification according to the reaction procedure.

### 2.5. Promoter Analysis and Protein Interaction Prediction of Cassava MeJAZ Family

TBtools-II was used to extract the 2000 bp upstream of the transcription initiation site of MeJAZ family members from the cassava genome sequence. The PlantCARE online tool (https://bioinformatics.psb.ugent.be/webtools/plantcare/html/) (accessed on 10 June 2025) was used to analyze cis-acting elements in the promoter region. TBtools-II and Excel software were used to calculate and visualize the predicted results of cis-acting elements. TBtools-II was used to extract candidate protein sequences from the cassava genome protein sequences, which were submitted to the Strings database (https://cn.string-db.org/) (accessed on 20 June 2025) for the alignment and prediction of protein interaction networks. Cytoscape 3.10.3 software was used to visualize the prediction results.

### 2.6. Method of Statistical Analysis of Data

It is imperative to note that all experiments were set up with no less than three biological replicates. The data are presented as the mean ± standard deviation. The statistical analysis was conducted utilizing SPSS Statistics 25.0 software, while graphical representation was achieved through the use of GraphPad Prism 10. For significant differences between treatments, a two-way ANOVA was employed to analyze the effects of different factors (e.g., MeJA treatment concentrations and AMF symbiotic status) and their interactions. The significance test employed Duncan’s multiple comparison method. The significance difference level was set at *p* < 0.05, and the extremely significant difference level was set at *p* < 0.01. Gene expression analysis was performed using the 2^−ΔΔCt^ method, and qRT-PCR data were normalized using the cassava *EF-1α* gene as an internal reference. Levels that were deemed to be of significance are indicated by asterisks. In the context of statistical analysis, the following symbols are used to denote the level of statistical significance: * *p* < 0.05, ** *p* < 0.01, ns indicates no significant difference.

## 3. Results

### 3.1. AM Symbiosis Promotes Growth and Development of Cassava and Increases Endogenous Hormone Levels

**Figure 1:**
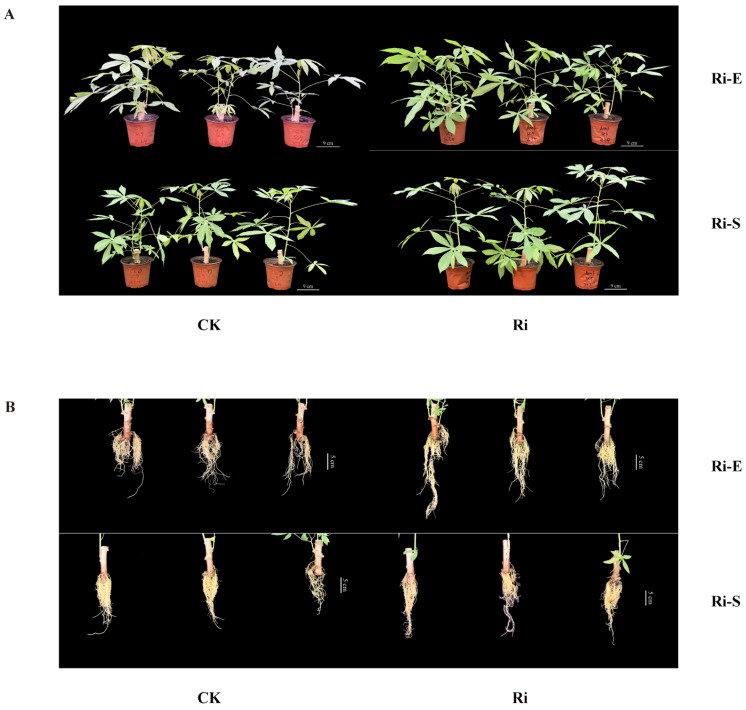
Effects of AMF on cassava growth phenotype. (**A**). Comparison of overall cassava seedling morphology between CK and AMF groups at early and stable symbiotic stages and (**B**). comparison of root morphology between CK and AMF groups at early and stable symbiotic stages.

In this study, the morphological characteristics of cassava in mycorrhizal and non-mycorrhizal treatments were detected. Mycorrhizal cassava (Ri, inoculated with AMF: *Rhizophagus irregularis*) and non-mycorrhizal cassava (CK, not inoculated with AMF) potted plants were sampled at the early and stable stages of symbiosis after inoculation with AMF (Figure 1). The influence of AMF on the growth of cassava seedlings was analyzed by measuring indicators such as plant height, stem thickness, fresh weight of the aboveground part, and root weight of the underground part. During the early stages of symbiosis, mycorrhizal cassava exhibited a slightly superior plant height compared to non-mycorrhizal cassava. During the stable symbiosis phase, mycorrhizal cassava had a significantly higher plant height (10.55%) than non-mycorrhizal cassava (Table A1). The fresh weight of the aboveground parts of mycorrhizal cassava was also significantly higher than that of non-mycorrhizal cassava in both periods, increasing by 31.02% and 13.91% at the two time points (Table A1). This indicates that inoculation with AMF has a certain promoting effect on the growth and development of the aboveground part of cassava after the establishment of the symbiotic relationship. To observe the effect of AMF inoculation on the underground parts of cassava plants, the plants were removed entirely from the pots in the early and stable stages of symbiosis after inoculation with AMF. Impurities on the root surface were gently washed with clean water, and the roots were dried with absorbent paper. Photos were taken to observe the phenotype (Figure 1B). We cut off the root completely and placed it under a balance to weigh the fresh weight of the underground root. Table A1 shows that the fresh weight of mycorrhizal cassava roots increased significantly by 23.59% in the early stage of symbiosis compared with the control group, and increased extremely significantly by 29.61% in the stable stage of symbiosis. This indicates that AMF can significantly affect the growth and development of the underground roots of cassava after symbiosis, especially in the early stage of symbiosis, and this effect increases over time.

**Figure 2:**
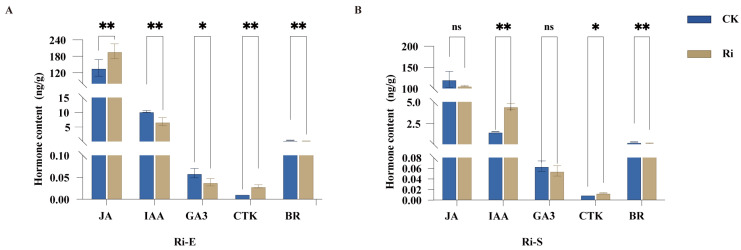
Effects of AMF on endogenous hormone levels in cassava root systems. (**A**). Endogenous hormone levels in cassava root systems during the early symbiotic stage and (**B**). Endogenous hormone levels in cassava root systems during the stable symbiosis stage. Quantitative comparison of jasmonic acid (JA), indoleacetic acid (IAA), gibberellin (GA3), cytokinin (CTK), and brassinolide (BR) in cassava plants inoculated with AMF at early and stable symbiotic stage. Bars represent mean ± standard error (n = 3 biological replicates). Asterisks indicate statistically significant differences between treatments (* *p* < 0.05, ** *p* < 0.01; ns: no significant difference. Student’s *t*-test).

Five hormones in young cassava roots, including Auxin (AUX), Gibberellin (GA), Cytokinin (CTK), Brassionsteroids (BR), and Jasmonic Acid (JA), were determined. Among them, the first four are recognized plant hormones. They all exist in plants in the form of small molecules and regulate the growth and differentiation of cells, promoting the growth and development of plants. JA is a fatty acid derivative that can regulate various important biological processes of plants, such as growth, development, and defense, and plays an important regulatory role in the growth and development processes of plant roots and the formation of tuberous roots. Through the detection of hormones in the young roots of cassava inoculated with AMF and uninoculated, it was found that the content of hormones in cassava after AMF inoculation was not a single effect. By regulating the changes in the content of different hormones, the growth of cassava seedlings can be promoted. The results of this study (Figure 2) show that, in the early symbiotic stage, the JA content in the mycorrhizal treatment group was significantly higher than that in CK (*p* < 0.01). The content of CTK in the mycorrhizal treatment group was extremely significantly higher than that in CK (*p* < 0.001). The content of IAA in the mycorrhizal treatment group was significantly lower than that in CK. The GA3 content in the mycorrhizal treatment group was significantly lower than that in CK (*p* < 0.05). The content of BR in the mycorrhizal treatment group was significantly lower than that in CK (*p* < 0.001). In the stable symbiotic stage, the content of IAA in the mycorrhizal treatment group was extremely significantly higher than that in the control group (*p* < 0.001); the content of CTK in the mycorrhizal treatment group was significantly higher than that in the control group (*p* < 0.05). The content of BR in the mycorrhizal treatment group was significantly lower than that in CK (*p* < 0.001). However, there was no significant difference in the contents of JA and GA3 between the mycorrhizal treatment group and the control group. The above results indicate that the effect of inoculation treatment on the content of plant hormones is time-dependent and hormone-specific, and the regulatory effects on various hormones vary at different growth stages.

### 3.2. GSEA Analysis of RNA-Seq Data Enriched Two Core Genes, MeJAZ6 and MeJAZ7

**Figure 3:**
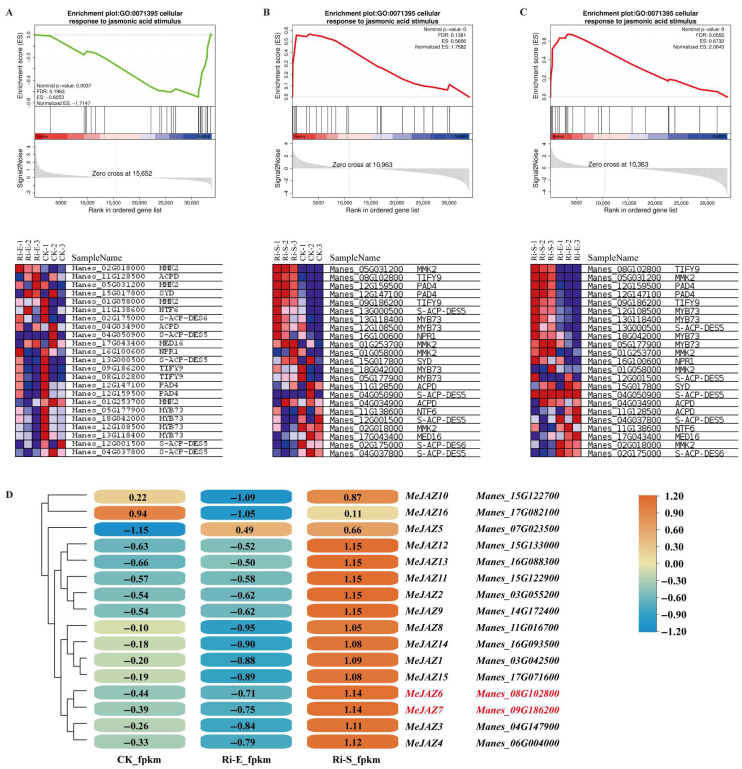
GSEA analysis and gene expression association analysis of GO gene set. (**A**–**C**) are GSEA (gene set enrichment analysis) results of GO:00071395 gene set, showing the enrichment score change trend of gene set in samples through broken line graph, and the expression pattern of corresponding genes in the lower heat map; (**D**) is the cluster heat map of expression quantity of *MeJAZ* family genes in different samples (CK, Ri-E, and Ri-S), indicating the change amplitude of gene expression quantity with color gradient, visually showing the expression correlation and pattern difference of genes under different conditions.

Based on published RNA-Seq data, this study focused on genes involved in the jasmonate signaling pathway for GSEA analysis. Figure 3A–C show GO enrichment (GO: 0071395) in response to the jasmonate signaling pathway in normal plants, AM early symbiosis plants, and AM symbiosis maintenance plants. During the AM symbiosis maintenance phase, we found that the enriched core genes included MMK2, PAD, MYB73, S-ACP-DES5, NPR1, and two *TIFY* genes. The two *TIFY* genes are members of the *MeJAZ* family, *Manes_08G102800* (*MeJAZ6*) and *Manes_09G186200* (*MeJAZ7*). The FPKM values of 16 members of the *MeJAZ* family were visualized and clustered by TBtools (Figure 3D). It was found that *MeJAZ* family members can respond to the AM symbiosis process, in which the expressions of the *MeJAZ6* and *MeJAZ7* genes were clustered into a cluster and the expressions of the *MeJAZ10* and *MeJAZ16* genes were also clustered into a cluster.

### 3.3. Analysis of Cis-Acting Elements in Promoter Region of MeJAZ Family

**Figure 4:**
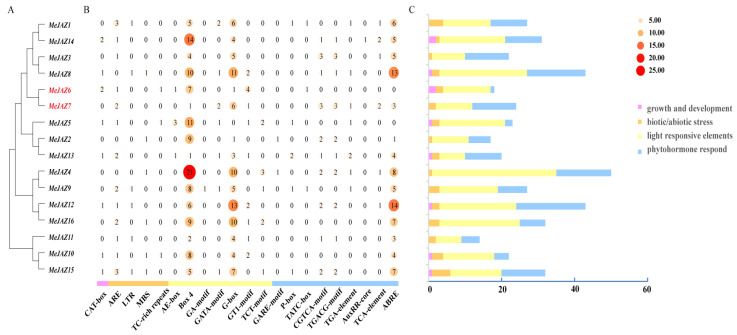
Analysis of cis-acting response elements in the promoter region of MeJAZ genes. (**A**): MeJAZ gene family evolutionary tree. (**B**): Heat map of the number of cis-acting elements in the promoter region of the *MeJAZ* genes. (**C**): Statistical bar chart of the four types of response elements of the MeJAZ gene promoter.

As can be seen from Figure 4, the promoter region of *MeJAZ* genes contains a variety of cis-acting response elements (Figure 4A–C), mainly including growth and development response elements, biotic or abiotic stress response elements, light response elements, and hormone response elements (Figure 4C). The maximum number of photoreactive elements is 250 (AE-box, Box 4, GA-motif, GATA-motif, G-box, GT 1-motif, and TCT-motif). The number of hormone response elements is 149, including 11 gibberellin response elements (GARE-motif, P-box, and TATC-box), 36 MeJA response elements (CGTCA-motif and TGACG-motif), 9 auxin response elements (TGA-element and auxRR-core), 8 salicylic acid response elements (TCA-element), and 85 abscisic acid response elements (ABRE). The developmental response element consists of 10 CAT-boxes. There are 36 stress response elements, including 21 anaerobic induction response elements (AREs), 6 low-temperature induction response elements (LTR), 6 drought induction response elements (MBS), and 3 defense and stress response elements (TC-rich repeats). *MeJAZ 2/4/5/8/10/15/16* members contain MBS elements in the MYB protein binding region involved in drought stress and the regulation of flavonoid biosynthesis genes, which also indicates that these genes play an important role in drought and flavonoid production and stimulus response (Figure 4B). In addition, there are 2 circadian, 22 WUN-motifs, and 72 MYB elements. *MeJAZ* gene family members have different numbers and types of hormone response elements, among which the *MeJAZ12* gene promoter has the most hormone response elements, up to 19, and the *MeJAZ7* gene promoter has 6 MeJA response elements, 1 auxin response element, 2 salicylic acid response elements, and 3 abscisic acid response elements, while the *MeJAZ6* gene promoter has only 1 hormone response element (TATC-box) (Figure 4B). These results suggest that the MeJAZ family plays an important role in plant growth and stress management, and different types of regulatory elements indicate that the MeJAZ family is involved in different cellular pathways.

### 3.4. Expression Pattern Analysis of MeJAZ Gene Family After AM Symbiosis

**Figure 5:**
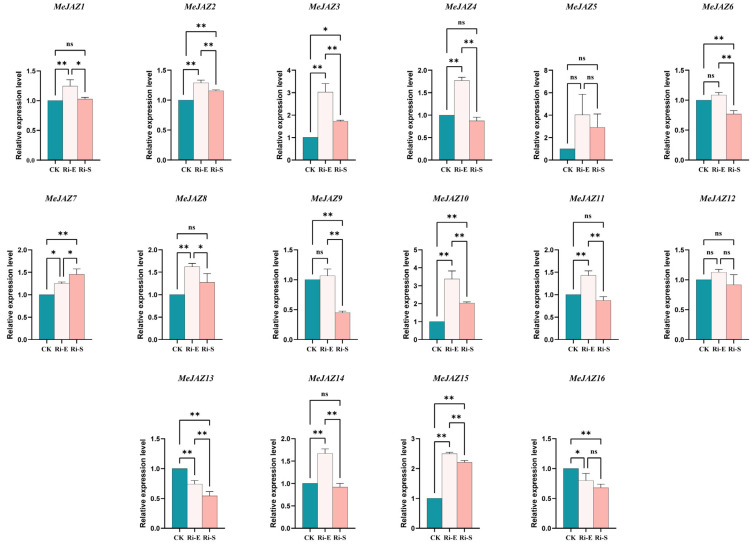
Expression analysis of MeJAZ family genes in different treatment groups. Each panel shows the relative expression of different MeJAZ genes in the control group (CK), Ri-E group, and Ri-S group, respectively. Ri-E: Early symbiotic stage. Ri-S: Stable symbiotic stage. Different color bars in the histogram represent corresponding groups, and the expression differences of each gene under different treatment conditions are presented through inter-group comparison. Bars represent mean ± standard error (n = 3 biological replicates). Asterisks indicate statistically significant differences between treatments (* *p* < 0.05, ** *p* < 0.01; ns: no significant difference. Two-way ANOVA).

The relative expression levels of each *MeJAZ* gene in the early and stable stages of mycorrhizal symbiosis were analyzed by real-time fluorescence quantitative PCR (qRT-PCR) technology, and statistical methods were used to analyze the differences between groups. The results indicated that the responses of different *MeJAZ* genes to AMF were significantly different (Figure 5). There were seven main expression patterns. (1) Five genes, namely *MeJAZ1/4/8/11/14*, were upregulated in the early stage of symbiosis. After entering the symbiotic stable period, their expression levels dropped to the same level as the control plants. (2) The expression levels of the four *MeJAZ2/3/10/15* genes were also upregulated in the early stage of symbiosis. Although the expression levels decreased during the stable period, they were still significantly higher than those of the control plants. (3) *MeJAZ7* was responded to both in the early and stable stages of mycorrhizal symbiosis, with its expression level continuously upregulated. (4) Two genes, *MeJAZ5* and *MeJAZ12*, did not receive a significant response during mycorrhizalization, and there was no significant difference in their expression levels. (5) Two genes, *MeJAZ6* and *MeJAZ9*, showed no difference from the control plants in the early stage of symbiosis, but their expression levels decreased during the stable symbiosis period. (6) *MeJAZ13* responded both in the early and stable stages of mycorrhizal symbiosis, with its expression level continuously downregulated. (7) *MeJAZ16* had a downregulated response expression in the early stage of symbiosis and maintained this level unchanged in the later stage. Expression pattern analysis indicated that *MeJAZ7* may continuously positively regulate the cassava mycorrhizalization process, and the *MeJAZ6/9/13/16* gene may continuously negatively regulate the cassava mycorrhizalization process. There were no significant changes in the *MeJAZ5* and *MeJAZ12* genes during the establishment of symbiosis between cassava and mycorrhizae. As the initiating response genes of the JA signaling pathway, the expression levels of multiple *MeJAZ* genes were upregulated and then downregulated, corresponding to the result that the content of endogenous jasmonic acid hormone in cassava increased and then decreased during the mycorrhizal infection process. Different members of the *MeJAZ* gene family may be involved in multiple reactions during the AMF symbiosis process, and they have a temporal sequence of symbiosis with AMF at different growth and development stages. The expression regulation of *MeJAZ* genes is dynamically adjusted along with the symbiotic process, undertaking different molecular functions and having a division of labor in responding to symbiotic signals. Arboreal mycorrhizal fungi significantly affect the relative expression levels of cassava *MeJAZ* gene family members, and this influence presents a complex regulatory pattern during the developmental stage, laying the foundation for further exploration of the molecular mechanism of cassava and AMF symbiosis and the function of *MeJAZ* genes.

### 3.5. Low Concentrations of Jasmonic Acid Can Eliminate and Inhibit the Promotion of AM Symbiosis on Cassava Growth and Development

By reviewing the literature and the gradient results of the pre-experiment, in this experiment, 50 µm/L (low concentration) and 100 µm/L (high concentration) of methyl jasmonate (MeJA) were sprayed to treat cassava leaves. The dilution and pre-spraying preparation methods of methyl jasmonate are referred to in the Materials and Methods section. Based on the previous cassava inoculation experiment, we found that mycelium began to form in the second week after AMF inoculation, and the initial stage of infection and symbiosis was reached in the third week. Therefore, we started to spray exogenous jasmonic acid from the beginning of the second week of inoculation (the 7th day), spraying once every 3 days, and taking samples at a time point of two weeks (i.e., the 21st and 36th days). The specific pot experiment treatment methods and sampling diagrams are shown in Figure S1.

Through phenotypic analysis after the continuous spraying of low-concentration (50 µm/L) jasmonic acid to SC9 cassava under normal growth (Figure S2), we found that after spraying 50 µm/L of jasmonic acid for 36 days, the fresh weight of the underground part of cassava was significantly increased (35.32%) compared with the control group without spraying. Sustained application of 50 µm/L of jasmonic acid inhibited stem diameter increase during normal growth, while synergizing or promoting an increase in the fresh weight of underground parts during growth (Figure S2, Table A2). Two-factor interaction analysis showed that the interaction between the continuous spraying of 50 µm/L of jasmonic acid and the two growth time points of cassava significantly affected the fresh weight of the underground part, *p* = 0.0019 (Table A2). This indicates that the timing of the stimulation of jasmonic acid had a positive regulatory effect on cassava root’s growth and development.

During the mycorrhizal cassava process, the continuous application of low-concentration (50 µm/L) jasmonic acid reduced the fresh weight of cassava underground and aboveground during the establishment of mycorrhizal symbiosis. However, there was no significant difference in mycorrhizal cassava’s plant height and stem diameter. During the symbiotic stable period, the fresh weight of underground parts decreased by 25.10% and that of aboveground parts decreased by 12.93% (Table A2). Two-factor interaction analysis showed that the continuous spraying of 50 µm/L of jasmonic acid and the mycorrhizalization of cassava had a significant interaction effect on promoting shoot fresh weight (*p* < 0.0001). In addition, the mycorrhizal maintenance period and the continuous spraying of low-concentration jasmonic acid also had a significant interaction effect on the fresh weight of underground parts, *p* ≤ 0.0001 (Table A2). We speculated that treating mycorrhizal cassava with a low concentration of methyl jasmonate may affect changes in jasmonate content in the body and delay the growth of plants.

**Figure 6:**
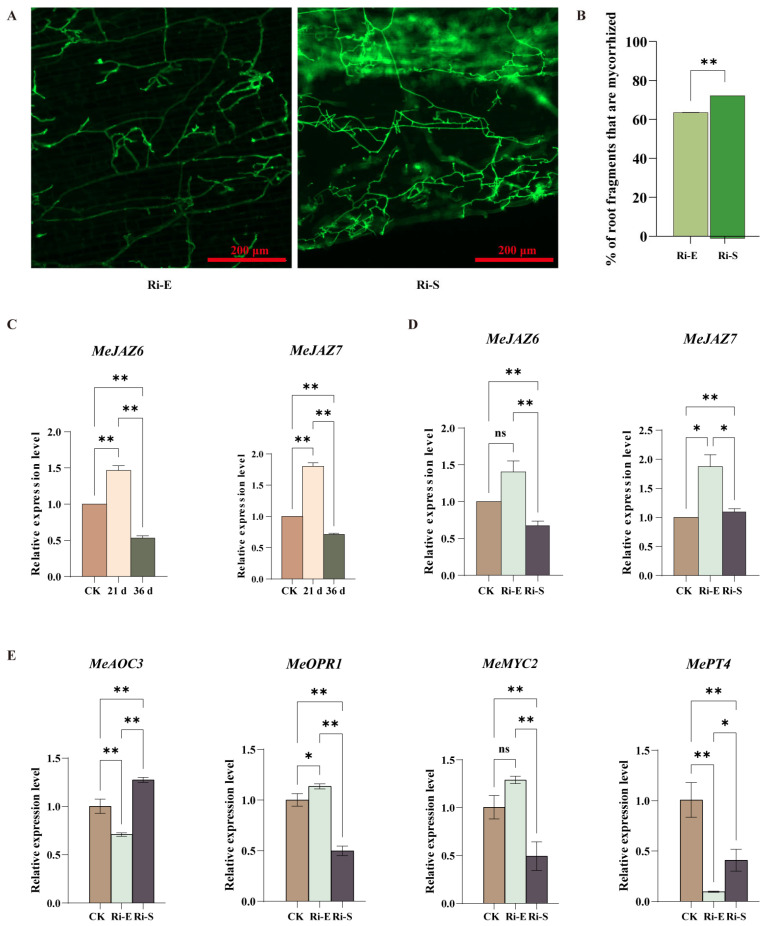
Effect of low concentration of jasmonic acid on symbiotic interaction between cassava and AM. (**A**). Microscopic observation of AMF symbiosis after treatment with low-concentration jasmonic acid. The green organelles indicate proliferating fungal hyphae. Pictures were taken at 20×, and scale bars indicate 200 µm. (**B**). Quantitative comparison of cassava planting rate. (**C**). Analysis of the expression pattern of the MeJAZ gene family in normal cassava treated with low concentration. (**D**). Analysis of the expression pattern of the MeJAZ gene family in mycorrhizal cassava treated with low concentration. (**E**). Expression levels of *MePT4*, *MeAOC3*, *MeOPR1*, and *MeMYC2* genes during the initial and maintenance stages of AM symbiosis. Bars represent mean ± standard error (n = 3 biological replicates). Asterisks indicate statistically significant differences between treatments (* *p* < 0.05, ** *p* < 0.01; ns: no significant difference. (**B**): Student’s *t*-test; (**C**–**E**): Two-way ANOVA).

Microscopic observation of cassava roots under continuous low-concentration jasmonic acid treatment (Figure 6A,B) showed that, compared with the control group, the infection rate of mycorrhizal fungi under low-concentration jasmonic acid treatment increased with time, and the hyphal distribution area and arbuscular root formation also increased with time. These results indicate that a low concentration of jasmonic acid increased the infection rate of mycorrhizal fungi and affected the growth and development of plants.

### 3.6. Expression Pattern Analysis of MeJAZ Gene Family in Cassava Treated with Low-Concentration Jasmonic Acid

To investigate the synergistic effect of MeJA and AMF on the expression of MeJAZ genes in cassava, we analyzed two sets of experimental data: when MeJA was applied only, among 16 *MeJAZ* genes, we found that except for *MeJAZ2, MeJAZ5*, and *MeJAZ6*, the expression of the other 13 *MeJAZ* genes was induced to be upregulated at the initial stage of symbiosis, and the expression level began to decrease at the stable stage of symbiosis, which also included the *MeJAZ2* gene (Figure 6C and Figure S3).

The *MeJAZ5*, *MeJAZ6*, *MeJAZ9,* and *MeJAZ12* genes were unchanged, the *MeJAZ13* and *MeJAZ16* genes were downregulated, and the *MeJAZ2* gene was upregulated in the initial stage of mycorrhizal symbiosis. *MeJAZ2* and *MeJA16* remained stable during the mycorrhizal symbiotic stabilization phase (Figure 6D). When low concentrations of exogenous MeJA were continuously sprayed, JA levels gradually accumulated in cassava roots, JAZ repressor protein released transcription factors to initiate downstream gene expression, and JAZ protein was simultaneously degraded by ubiquitination. JA response genes, JAZs, increased and then decreased. *MeJAZ13* and *MeJAZ16* were the first genes to be downregulated during mycorrhizal infection, suggesting that these two genes responded to mycorrhizal infection first. Compared with normal plants, *MeJAZ2* remained stable after induced upregulation, suggesting that *MeJAZ2* may be involved in various regulatory processes during mycorrhizalization.

In the context of a minimal concentration of methyl jasmonate (MeJA), there was a substantial inhibition of the expression of pivotal genes associated with AM symbiosis. The expression level of *MePT4*, a marker gene for AM symbiosis, was significantly downregulated, indicating that low concentrations of MeJA might interfere with the normal establishment of the AM symbiosis system. Among JA biosynthesis-related genes, *MeAOC3* was suppressed at the early stage of symbiosis, but its expression recovered and was re-upregulated at the stable stage of symbiosis. This suggests the presence of a phased feedback mechanism for this gene. Another synthetic gene, *MeOPR1*, was suppressed primarily in the early stages of symbiosis. Furthermore, *MeMYC2*, a pivotal transcription factor in the JA signaling response, continuously decreased throughout symbiosis. This finding suggests that low concentrations of MeJA may interfere with JA-mediated signal transduction, consequently affecting the establishment and maintenance of AM symbiosis (Figure 6E).

In conclusion, *MeJAZ* gene expression in cassava is regulated by time and gene specificity, and AMF can cooperate with MeJA to remodel *MeJAZ* gene expression patterns, which plays an important role in the regulation of *MeJAZ* gene expression in cassava in response to jasmonic acid signals and mycorrhizal symbiosis, which provides a basis for understanding the molecular mechanism of the interaction between the JA signal pathway and mycorrhizal fungi in cassava.

### 3.7. Promotion of Fresh Weight Accumulation of Cassava by High-Concentration Jasmonic Acid Synergistic AM Symbiosis Process

During normal growth, the continuous exogenous spraying of high-concentration (100 µm/L) jasmonic acid was found to promote an increase in the fresh weight of the underground and aboveground parts of mycorrhizalized cassava (Figure S4), which increased by 32.55% and 40.94%, respectively, on the 21st day, and increased by 55.10% and 24.30%, respectively, on the 36th day (Table A3). But this had no significant effect on plant height and stem diameter. Two-factor interaction analysis showed that the interaction of mycorrhizal cassava treated with 100 µm/L or MeJA at two time points mainly promoted the fresh weight of underground parts and produced significant differences (Table A3).

During the mycorrhizal process of cassava, the continuous exogenous application of 100 µm/L of jasmonic acid increased the fresh weight of shoots by 41% at the initial stage of mycorrhizal symbiosis, but had no effect on plant height and stem diameter. However, the continuous spraying of exogenous high-concentration jasmonic acid had no effect on the plant height, stem diameter, and fresh weight of cassava in the stable stage of mycorrhizal symbiosis (Table A3, Figure S4). Two-factor interaction analysis showed that the continuous spraying of 100 µm/L of jasmonic acid and the mycorrhizalization of cassava had a significant interaction effect on promoting shoot fresh weight (*p* = 0.0093). Among them, the interaction between the mycorrhizal establishment stage and the continuous spraying of high-concentration of jasmonic acid on the fresh weight of underground biomass was significant, *p* = 0.0316, while the effect of the interaction between the mycorrhizal maintenance stage and the continuous spraying of high-concentration of jasmonic acid on the fresh weight of underground biomass was extremely significant, *p* = 0.0009 (Table A3).

**Figure 7:**
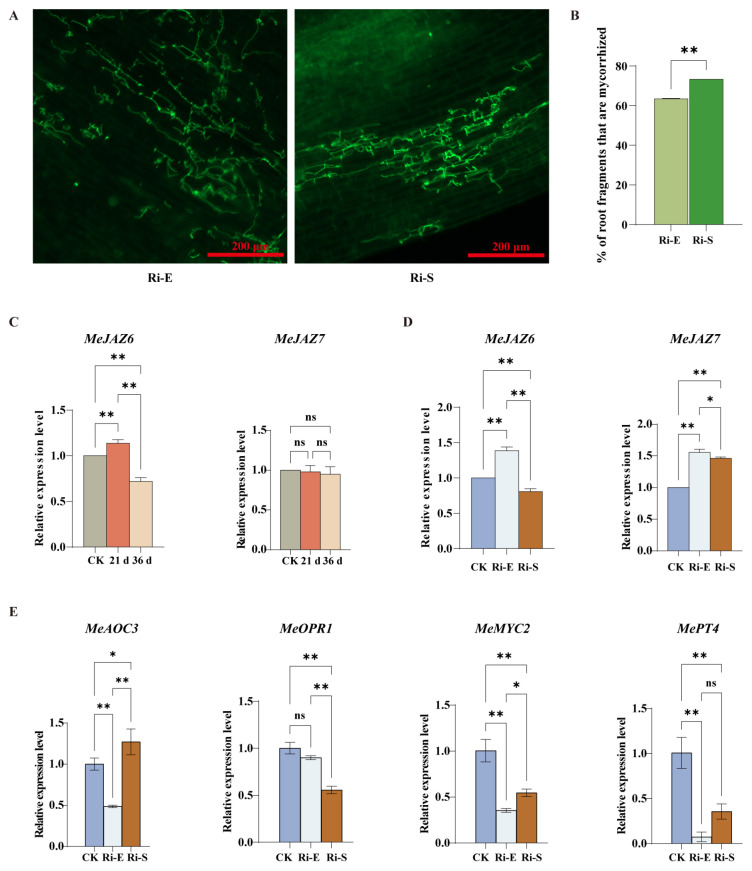
Effect of high concentration of jasmonic acid on symbiotic interaction between cassava and AM. (**A**). Microscopic observation of AMF symbiosis after treatment with high-concentration jasmonic acid. The green organelles indicate proliferating fungal hyphae. Pictures were taken at 20×, and scale bars indicate 200 µm. (**B**). Quantitative comparison of cassava planting rate. (**C**). Analysis of the expression pattern of the MeJAZ gene family in normal cassava treated with high concentration. (**D**). Analysis of the expression pattern of the MeJAZ gene family in mycorrhizal cassava treated with high concentration. (**E**). Expression levels of *MePT4*, *MeAOC3*, *MeOPR1*, and *MeMYC2* genes during the initial and maintenance stages of AM symbiosis. Bars represent mean ± standard error (n = 3 biological replicates). Asterisks indicate statistically significant differences between treatments (* *p* < 0.05, ** *p* < 0.01; ns: no significant difference. (**B**): Student’s *t*-test; (**C**–**E**): Two-way ANOVA).

Microscopic observation of cassava roots under continuous high-concentration jasmonic acid treatment (Figure 7A,B) showed that compared with the control group, the mycorrhizal infection rate increased with time under high-concentration jasmonic acid treatment, but the hypha distribution area and arbuscular root formation decreased compared with the control group. Some studies showed that a low concentration of jasmonic acid once a week could effectively stimulate the formation of mycorrhizal fungi, while shortening the treatment time and increasing the concentration had the opposite result, decreasing the infection rate of mycorrhizal fungi. This indicates that a high concentration of JA increased the infection rate of mycorrhizal fungi, but decreased the distribution of hyphae in roots, thus affecting the growth and development of plants.

### 3.8. Expression Pattern Analysis of MeJAZ Gene Family in Cassava Treated with High-Concentration Jasmonic Acid

The regulation of *MeJAZ* gene expression in cassava under the synergistic effect of continuous high-concentration jasmonic acid (100 μmol/L MeJA) and AMF treatment was analyzed. The results showed (Figure 7C,D and Figure S5) that *MeJAZ2/3/8/15* was positively regulated at high concentrations (Figure S5A) and *MeJAZ1/4/12/13/14/16* was negatively regulated at high concentrations when MeJA was applied only (Figure S5B). *MeJAZ6, MeJAZ9*, and *MeJAZ10* also showed diverse expression patterns after cassava was treated with exogenous high-concentration MeJA. Compared with a low concentration of jasmonic acid, JA accumulation in cassava roots was faster after high-concentration jasmonic acid treatment, and the downstream response of the JA signaling pathway was initiated. *MeJAZ1* and *MeJAZ10* were downregulated earlier, *MeJAZ1* remained stable after downregulation, and *MeJAZ10* was upregulated to a normal level later.

The *MeJAZ* gene expression pattern in the mycorrhizal roots of cassava treated with a high concentration of jasmonic acid was changed compared with that in normal plants (Figure 7D and Figure S5B). For example, *MeJAZ1/4/10/11/14* was upregulated at the initial stage of mycorrhizal symbiosis, downregulated after entering the stable stage of symbiosis, and then *MeJAZ1/4/11/14* returned to a normal level. *MeJAZ7* and *MeJAZ10* remained upregulated overall. It should be noted that the *MeJAZ6* and *MeJAZ9* genes were upregulated and downregulated, respectively, at the early stage of symbiosis, and the *MeJAZ5* and *MeJAZ8* genes were upregulated in the maintenance stage after high-concentration jasmonic acid treatment, compared with control plants and plants treated with a low concentration of jasmonic acid (the *MeJAZ6* and *MeJAZ9* genes were not induced in the initial stage; the *MeJAZ8* and *MeJAZ5* genes had no significant change in the maintenance stage). The expression of AM-symbiosis-related genes was significantly suppressed at elevated concentrations of MeJA. The expression of *MePT4* was found to be significantly inhibited, thus indicating that excessive JA may potentially impede the formation of functional mycorrhizal structures. *MeAOC3*, a pivotal enzyme in JA biosynthesis, was found to be inhibited in the early stage of symbiosis, yet subsequently induced in the stable stage of symbiosis. This finding suggests that *MeAOC3* may achieve late compensatory expression through a feedback mechanism under elevated JA level conditions. However, *MeOPR1* was continuously inhibited during the symbiotic phase, particularly during the stable phase. Furthermore, *MeMYC2* expression was found to be downregulated significantly at elevated MeJA concentrations, suggesting that excessive JA signaling may trigger negative regulatory mechanisms and inhibit JA response transcriptional networks, thereby interfering with the maintenance and functional perfection of AM symbiotic systems (Figure 7E).

The results indicate that the regulation of *MeJAZ* gene expression by high-concentration jasmonic acid treatment was affected by gene characteristics and timing stages, and AMF could cooperate with jasmonic acid to change *MeJAZ* gene expression pattern, which played an important role in the molecular mechanism of cassava participating in jasmonic acid signal response and mycorrhizal symbiosis interaction, providing data support for further analysis of related molecular regulatory pathways.

### 3.9. PPI Predicts the Interaction of Candidate JAZ Proteins with Known Key Factors of SYM Signaling Pathway

**Figure 8:**
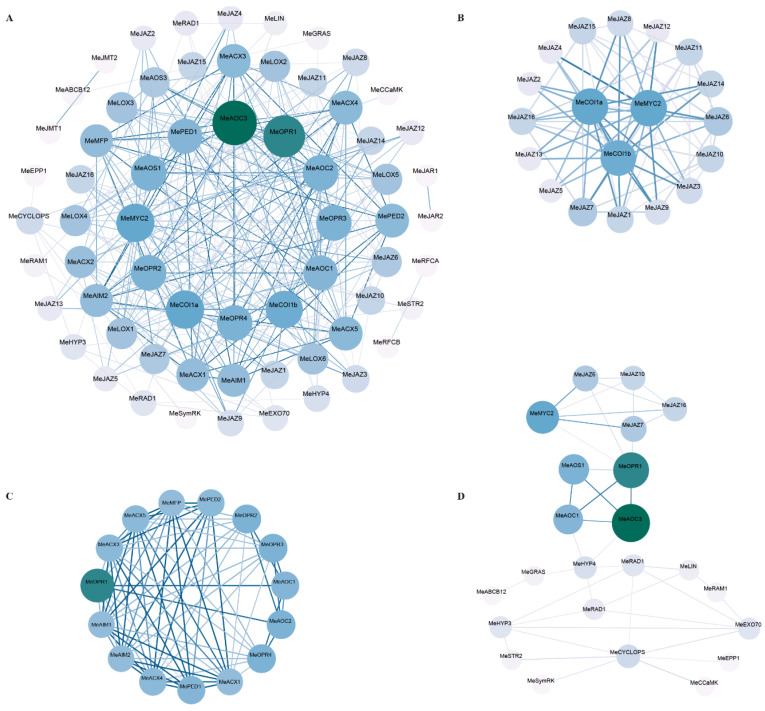
PPI predicts the interaction between JA biosynthesis, JA signal response protein, and AM symbiotic signal key factors. The overall protein interaction prediction results. (**B**–**D**) are subnetworks of (**A**). (**B**) The subnetworks with the highest scores filtered by the Cytoscape 3.10.3 MCODE plug-in (parameters set to default values). (**C**) The predicted interaction among MeCOI1, MeJAZ, and MeMYC2, key proteins of JA signaling pathway. (**D**) The prediction of interactions between candidate MeJAZ proteins, JA biosynthesis key proteins, and symbiotic signaling pathway key proteins. Darker and larger nodes represent a higher number of protein interactions, while darker edges indicate stronger interactions between nodes.

A protein interaction network was constructed using the Strings database to analyze the interactions of 14 proteins in the critical symbiotic (SYM) signaling pathway in cassava with core members of the JA biosynthesis and signaling processes (Figure 8). Figure 7A shows the overall protein interaction prediction results, and Figure 8B–D show the subnetworks. Figure 8A,B show the subnetworks with the highest scores (parameters set to default values) screened by the MCODE plug-in of the Cytoscape 3.10.3 software, which are important members of JA biosynthesis. Figure 7C shows the predicted interaction among COI1, JAZ, and MeMYC2, key proteins of the JA signaling pathway. Figure 8D shows interaction predictions among candidate MeJAZ proteins, JA biosynthesis key proteins, and symbiotic signaling pathway key proteins. The results show that the MeAOC3 and MeOPR1 proteins of the JA synthesis pathway are major core proteins in the prediction results, and they not only have strong interactions (Figure 8A), but also serve as bridge proteins between the JA signaling pathway and SYM pathway proteins (Figure 8D). MeOPR1 interacts with MeJAZ6, MeJAZ7, and MeMYC2 protein prediction, MeAOC3 and MeAOC1 interact with MeHYP3 protein prediction, and MeHYP3 interacts with GRAS, RAD1, and other symbiotic factors prediction (Figure 8D). PPI prediction suggested that JAZ protein might be involved in AM symbiosis through a hierarchical regulation model centered on jasmonic acid signals, through signal perception (MeCOI1, MeJAZ, and MeMYC2), metabolic synthesis (MeAOS/MeAOC/MeOPR), and downstream AM symbiotic factor response (MeGRAS/MeHYP).

## 4. Discussion

In recent years, more and more attention has been paid to the effects of AMF on plant growth, yield, quality, and stress tolerance, and it is expected that AMF can be used as a biological fertilizer to achieve sustainable agriculture and food security. An in-depth understanding of the molecular mechanisms and regulation of AMF symbiosis is a prerequisite for achieving this goal.

This study found that the symbiosis of AMF and cassava significantly promoted the growth and development of cassava, especially in the root growth of underground parts. Mycorrhizal cassava was superior to non-mycorrhizal cassava in root weight, lateral root weight, and root length. The upregulation of the phosphate transporter *MePT4* gene induced by AMF was also detected in this study (Figure S6). The infection rate of arbuscular rhizomycetes increased under microscope . This result is consistent with previous studies suggesting that AMF promote plant growth by improving the plant uptake of nutrients and water [43,44]. After AMF form a symbiotic relationship with plant roots, the hyphal network can expand the absorption range of plant roots and increase the efficiency of obtaining nutrients such as phosphorus and nitrogen [44]. In addition, AMF symbiosis may also promote plant growth by regulating plant hormone levels [45,46].

When mycorrhizal fungi form symbiosis with plant roots, they can significantly change the hormone balance in the host body. Arbuscular mycorrhizal fungi can promote plant root growth and induce plant molecular and biochemical reactions after infecting host plants [45,47,48]. In this study, AMF symbiosis significantly affected the endogenous hormone levels of cassava, especially changes in jasmonic acid (JA), cytokinin (CTK), and auxin (IAA). These hormones play key regulatory roles in plant growth and development. For example, JA plays an important role in plant root growth and defense responses [49], while CTK is primarily involved in cell division and differentiation [50]. In this study, changes in JA and CTK contents after mycorrhizal treatment were closely related to the promotion of root growth of cassava, indicating that AMF may affect plant growth by regulating hormone levels. Liu et al. also pointed out that AMF can enhance plant nutrient absorption capacity and stress resistance by regulating the plant hormone network, which further supports the conclusion of this study [48].

AMF can regulate the plant defense system through salicylic acid and jasmonic acid signaling pathways during the process of forming mycorrhiza by infecting plant roots, thus improving plants’ ability to cope with biotic and abiotic stresses [51,52]. The jasmonic acid signal transduction pathway plays an important role in the process of AMF infection host mycorrhizal formation [53]. AMF infection induced the early accumulation of some defense-related hormones such as kasmonic acid (JA), abscisic acid (ABA), and salicylic acid (SA) in roots [35]. This hormonal fluctuation is not an isolated event, but a key strategy of the mycorrhizal symbiont to coordinate plant development and stress resistance. For example, mycorrhizal plants promote JA precursor OPDA production by upregulating JA synthesis genes (such as *LOX* and *AOS*), activating downstream defense pathways [36]. This increase in JA is spatiotemporal-specific—JA levels usually return to baseline late in symbiosis establishment, suggesting that mycorrhizal fungi may avoid over-defense responses by inhibiting symbiosis through negative feedback mechanisms [54].

By GSEA analysis of RNA-Seq data, two core genes, *MeJAZ6* and *MeJAZ7*, belonging to the TIFY gene family in the JA signal transduction pathway, were enriched in this study. The MeJAZ gene family plays an important role in plant growth and development and stress response [40]. Analysis of cis-acting elements in the promoter region revealed that MeJAZ gene family members contain multiple hormone response elements and stress response elements, suggesting that they may be involved in regulating multiple signaling pathways [55]. Furthermore, the analysis of MeJAZ gene family expression patterns during AMF symbiosis showed significant differences among members during the initial and stable stages of symbiosis, which may be related to their functional division during symbiosis.

In this study, we also examined the expression levels of the JA biosynthesis-related genes *MeAOC3* and *MeOPR1* and the JA signal responsive gene *MeMYC2* (Figure S4). The results showed that, compared with the control group, AM symbiosis could strongly induce the expression of the *MeAOC3* gene in the initial and maintenance stages, and the expression level was more prominent in the stable symbiotic stage. *MeOPR1* and *MeMYC2* were upregulated in the early phase of AM symbiosis, but *MeOPR1* did not respond during the AM symbiosis maintenance phase, while *MeMYC2* expression was suppressed. This suggests that the JA signaling pathway is necessary for symbiosis establishment and maintenance, revealing the necessity of the JA pathway for symbiotic maintenance [51]. The difference in gene expression pattern was related to gene function and experimental treatment. In the early stage of AM symbiosis, genes were widely upregulated by activating hormone signal pathways such as jasmonic acid, and in the maintenance stage of AM symbiosis, gene expression was differentially regulated by different metabolic pathway branches. The strong induction of *MeAOC3* by AMF suggests that *MeAOC3* may also regulate AM symbiosis alone. Further investigation can be combined with gene function verification.

The effects of exogenous jasmonic acid on AMF symbiosis were also discussed. The results showed significant differences between low and high concentrations of jasmonic acid on cassava growth and AMF symbiosis. Under normal growth conditions, a low concentration of jasmonic acid promoted the growth of the underground part of cassava, but inhibited the accumulation of the fresh weight of the aboveground part and underground part during mycorrhizalization. This may have been related to the effect of jasmonic acid on the mycorrhizal infection rate. Microscopic observation showed that a low concentration of jasmonic acid treatment increased the infection rate of mycorrhizal fungi, but could affect the growth and development of plants by changing the JA level in plants, which is similar to previous studies [56].

In contrast, high concentrations of jasmonic acid significantly increased shoot fresh weight at the initial stage of mycorrhizal symbiosis, but the effects on the mycorrhizal infection rate and hyphal distribution were more complex. High concentrations of jasmonic acid may affect AMF symbiosis by accelerating the activation of the JA signaling pathway and altering the expression pattern of the MeJAZ gene [49]. Recent studies have also shown that the effects of jasmonic acid concentration and treatment time on plant–microorganism interaction are dose-dependent and time-dependent. For example, low concentrations of jasmonic acid can promote symbiotic relationships between plants and beneficial microorganisms, while high concentrations may inhibit symbiotic processes [57,58]. This is consistent with the results of this study, which further emphasizes the complex regulatory role of jasmonic acid in plant symbiosis. The expression of *MePT4*, a key gene of AM symbiosis, was strongly suppressed by JA treatment at both concentrations. The expressions of *MeAOC3* and *MeOPR1*, genes related to JA biosynthesis, were suppressed in the initial and maintenance stages of AM symbiosis, respectively, but *MeAOC3* was upregulated in the late stage of AM symbiosis. The expression level of *MeMYC2*, a JA signaling response gene, was also significantly suppressed. Furthermore, the expression levels of JAZ genes and the effects on growth in the cassava AMF symbiotic system exhibited significant variations between low and high concentrations of jasmonic acid. The expressions of JAZ7 and JAZ10, induced by low concentrations of jasmonic acid, were positively correlated with fresh weight accumulation. Furthermore, mycorrhizal symbiosis enhanced the growth promotion effect of jasmonic acid. However, under high-jasmonic-acid-concentration conditions, the sustained high expression of JAZ7 and JAZ10 genes resulted in growth inhibition, and mycorrhizal symbiosis exhibited partial efficacy in mitigating these adverse effects, though a complete reversal proved challenging. The results of this study indicate that the regulation of the JAZ genes is synergistically influenced by the concentration of jasmonic acid and the symbiotic state during the response to jasmonic acid signals.

Based on PPI prediction analysis, we hypothesized that JAZ protein may be involved in AM symbiosis through interaction with key proteins in JA biosynthesis and signal transduction. For example, AOC3 and OPR1 proteins in the JA synthesis pathway potentially interact with key factors in symbiotic signaling pathways [59]. This cross-pathway protein interaction network provides new insights into the molecular regulation of AMF symbiosis.

Jasmonic acid showed functional contradiction in mycorrhizal interactions [60]. This paradox can be explained by a model of precise temporal regulation of hormones [61]: early transient increases in JA are used to select compatible fungal partners, while late JA signal decay creates a microenvironment conducive to nutrient exchange. In this molecular mechanism, JAZ protein degradation mediated by JA receptor COI1 may release transcription factors (such as MYC2), which, in turn, regulate the expression of symbiotic related genes (such as RAM1 and STR) . The differential responses of different MeJAZ members to AMF and MeJA and the prediction of protein interactions in this study support this mechanism, in which MeJAZ6 and MeJAZ7 may be in a more central regulatory position.

Mycorrhizal fungi significantly enhance plant resistance to biotic and abiotic stresses by remodeling JA signals [62,63]. In terms of disease resistance, AMF pre-infection induces mycorrhiza-induced resistance (MIR), which relies on the JA/ET signaling pathway rather than the SA pathway [64]. For example, when pathogenic fungi attacked mycorrhizal tomato plants, the expression level of the JA response gene PI-II was increased more than three times compared with that of non-mycorrhizal plants [65]. In this study, MeJAZ gene promoters were rich in stress response elements (such as MBS, LTR, and TC-rich repeats), and multiple MeJAZ genes (such as *MeJAZ2*, *MeJAZ4*, etc.) were induced by AMF and MeJA, suggesting that they may be involved in integrating symbiotic signals and environmental stress responses [66]. In drought resistance, mycorrhizal fungi can reduce water loss by enhancing JA-mediated stomatal closure response, while activating JA-regulated antioxidant enzymes such as SOD and POD to alleviate oxidative damage [67]. In this study, AMF significantly promoted the root development of cassava, combined with the activation of the JA signaling pathway, which may enhance cassava’s stress tolerance. This synergy of mycorrhiza–JA pathways provides an important basis for developing eco-friendly stress tolerance strategies.

Recent studies have also revealed complex interactions between jasmonate and symbiotic signaling pathways. For example, Wang et al. found that key transcription factors in the JA signaling pathway can interact with key factors in the symbiotic signaling pathway to regulate the symbiotic relationship between plants and mycorrhizal fungi [44]. The PPI prediction results of this study further support this view, suggesting that JAZ protein may be involved in regulating AM symbiosis through interaction with key proteins in JA biosynthesis and signal transduction. This cross-pathway interaction model provides a new direction for further understanding the molecular regulation mechanism of AMF symbiosis.

This study will systematically reveal the central role and molecular mechanism of JAZ protein in regulating AMF symbiosis establishment and homeostasis maintenance in cassava, an important food crop, and provide an important case for further understanding the dual regulatory role of plant hormones (especially JA) in symbiosis establishment. The research results will also provide a solid theoretical basis and genetic resources for the future to accurately regulate the symbiotic efficiency of cassava mycorrhizal fungi through molecular design or genetic improvement, cultivate a new germplasm of “efficient-symbiotic-type” cassava, reduce fertilizer dependence and enhance environmental adaptability, and finally serve the sustainable production of resource-saving and environment-friendly cassava.

## 5. Conclusions

This study systematically revealed the key regulatory role of JAZ proteins during the initial and stable stages of cassava–AM symbiosis and elucidated the synergistic molecular mechanism between JA signaling and AMF interaction. The results demonstrated that members of the JAZ gene family, particularly MeJAZ7, exhibited stage-specific expression patterns throughout the symbiotic process, suggesting their potential function as central regulatory nodes that coordinate JA-mediated hormonal signaling with symbiotic pathway integration. PPI predictions further corroborated the hypothesis that JAZ proteins may interact with key components of both the JA biosynthesis pathway and the SYM signaling pathway, thereby mediating cross-talk across signaling modules and forming a multilayered regulatory model of “JA signaling–JAZ regulation–symbiosis pathway.” This study contributes to our understanding of the dual regulatory roles of phytohormones in the establishment and maintenance of plant–microbe symbiosis. It provides valuable theoretical insights and gene resources for the molecular breeding of cassava and other food crops, with a view to improving nutrient use efficiency and environmental adaptability.

## Data Availability

The data presented in this study are available in this article or Supplementary Material.

## Supplementary Materials

The following supporting information can be downloaded at: https://www.mdpi.com/article/10.3390/jof11080601/s1, Figure S1. The treatment method of the pot experiment and the schematic diagram of two samplings. Figure S2. Growth phenotype of cassava seedlings treated with low concentration of jasmonic acid. Figure S3. Analysis of the expression pattern of the MeJAZ gene family in cassava under low-concentration jasmonic acid treatment. Figure S4. Growth phenotype of cassava seedlings treated with high concentration of jasmonic acid. Figure S5. Analysis of the expression pattern of the MeJAZ gene family in cassava under high-concentration jasmonic acid treatment. Figure S6. Expression of *MePT4*, *MeAOC3*, *MeOPR1*, and *MeMYC2* genes in AM symbiosis initial stage and maintenance stage. Table S1. Primer sequences used in this study. Table S2. Formula of cassava modified low phosphorus Hoagland nutrient solution. Table S3. Reagents for qPT-PCR.

## Appendix A

**Table A1:** Effects of mycorrhizal treatment and untreated treatment on cassava growth at different stages.

	Parameter	CK Average	AMF Average	Ratio (%)	*p* Value Summary	Adjusted *p* Value
Early symbiotic stage	Plant height (cm)	22.00 ± 0.55	21.71 ± 0.06	−1.32	ns	0.9338
	Stem thick (mm)	4.33 ± 0.12	4.43 ± 0.19	2.31	ns	0.7755
	Aboveground fresh weight (g)	6.84 ± 0.29	9.97 ± 0.04	**31.01**	******	**0.0001**
	Belowground fresh weight (g)	1.99 ± 0.22	2.45 ± 0.14	**23.49**	*****	**0.0166**
Stable symbiotic stage	Plant height (cm)	24.04 ± 0.42	26.58 ± 1.56	**10.55**	*****	0.0353
	Stem thick (mm)	4.80 ± 0.14	4.8 ± 0.14	0.00	ns	>0.9999
	Aboveground fresh weight (g)	11.59 ± 0.40	13.20 ± 0.22	**13.90**	******	**0.0007**
	Belowground fresh weight (g)	2.75 ± 0.07	3.56 ± 0.03	**29.61**	******	**0.0006**

Asterisks indicate statistically significant differences between treatments * *p* < 0.05, ** *p* < 0.01; ns: no significant difference.

**Table A2:** The influence of continuous exogenous spraying of low-concentration jasmonic acid on growth at different periods.

		Parameter	J0 (MeJA 0 μm/L)	J50 (MeJA 0 μm/L)	Ratio ((J50-J0)/J0)	Two-Way ANOVA (J0 vs. J50)	*p* Value for Factor Interaction (E vs. M & J0 vs. J50)	*p* Value Summary	*p* Value for Factor Interaction (CK vs. Ri &J0 vs. J50)	*p* Value Summary
CK	21 d	Plant height (cm)	22 ± 0.67	19.37 ± 2.43	−0.12	ns				
		Stem thick (mm)	4.33 ± 0.15	3.97 ± 0.31	−0.08	ns				
		Aboveground fresh weight (g)	6.84 ± 0.36	7.71 ± 1.01	0.13	ns				
		Belowground fresh weight (g)	1.99 ± 0.27	2.04 ± 0.15	0.03	ns				
	36 d	Plant height (cm)	24.04 ± 0.51	21.17 ± 1.89	−0.12	ns	0.8969	ns		
		Stem thick (mm)	4.8 ± 0.17	4.37 ± 0.31	−0.09	ns	0.8196	ns		
		Aboveground fresh weight (g)	11.59 ± 0.5	12.28 ± 1.03	0.06	ns	0.8497	ns		
		Belowground fresh weight (g)	2.75 ± 0.09	3.72 ± 0.16	**0.35**	******	**0.0019**	******		
Ri	Ri-E	Plant height (cm)	21.71 ± 0.08	20.7 ± 1.45	−0.05	ns			0.3657	ns
		Stem thick (mm)	4.43 ± 0.23	4.2 ± 0.17	−0.05	ns			0.6195	ns
		Aboveground fresh weight (g)	8.96 ± 0.05	8.87 ± 0.2	−0.01	ns			0.1641	ns
		Belowground fresh weight (g)	2.45 ± 0.17	1.67 ± 0.22	**−0.32**	******			**0.0081**	******
	Ri-S	Plant height (cm)	25.91 ± 1.08	23.73 ± 0.66	−0.08	ns	0.3203	ns	0.6246	ns
		Stem thick (mm)	4.57 ± 0.51	4.6 ± 0.17	0.01	ns	0.4733	ns	0.2459	ns
		Aboveground fresh weight (g)	13.2 ± 0.27	11.49 ± 0.01	**−0.13**	******	**<0.0001**	******	**0.0075**	******
		Belowground fresh weight (g)	3.43 ± 0.18	2.57 ± 0.16	**−0.25**	******	0.7315	ns	**<0.0001**	******

Asterisks indicate statistically significant differences between treatments ** *p* < 0.01; ns: no significant difference.

**Table A3:** The influence of continuous exogenous spraying of high-concentration jasmonic acid on growth at different periods.

		Parameter	J0 (MeJA 0 μm/L)	J100 (MeJA 100 μm/L)	Ratio ((J100-J0)/J0)	Two-Way ANOVA (J0 vs. J100)	*p* Value for Factor Interaction (E vs. M & J0 vs. J100)	*p* Value Summary	*p* Value for Factor Interaction (J0 vs. J100 & CK vs. Ri)	*p* Value Summary
CK	21 d	Plant height (cm)	22 ± 0.67	21.33 ± 2.24	−0.03	ns				
		Stem thick (mm)	4.33 ± 0.15	3.97 ± 0.42	−0.08	ns				
		Aboveground fresh weight (g)	6.84 ± 0.36	9.63 ± 0.59	**0.41**	******				
		Belowground fresh weight (g)	1.99 ± 0.27	2.63 ± 0.17	**0.32**	*****				
	36 d	Plant height (cm)	24.04 ± 0.51	23.7 ± 1.79	−0.01	ns	0.8534	ns		
		Stem thick (mm)	4.8 ± 0.17	4.87 ± 0.15	0.01	ns	0.1717	ns		
		Aboveground fresh weight (g)	11.59 ± 0.5	14.42 ± 0.79	**0.24**	******	0.9614	ns		
		Belowground fresh weight (g)	2.75 ± 0.09	4.26 ± 0.39	**0.55**	******	**0.0182**	*****		
Ri	Ri-E	Plant height (cm)	21.71 ± 0.08	22.36 ± 0.87	0.03	ns			0.3871	ns
		Stem thick (mm)	4.43 ± 0.23	4.17 ± 0.15	−0.06	ns			0.7489	ns
		Aboveground fresh weight (g)	8.96 ± 0.05	12.61 ± 0.06	**0.41**	******			0.0651	ns
		Belowground fresh weight (g)	2.45 ± 0.17	2.5 ± 0.18	0.02	ns			**0.0316**	*
	Ri-S	Plant height (cm)	25.91 ± 1.08	25.17 ± 1.35	−0.03	ns	0.2461	ns	0.7886	ns
		Stem thick (mm)	4.57 ± 0.51	4.6 ± 0.3	0.01	ns	0.451	ns	0.9301	ns
		Aboveground fresh weight (g)	13.2 ± 0.27	14.08 ± 1.38	0.07	ns	**0.0093**	******	0.0804	ns
		Belowground fresh weight (g)	3.43 ± 0.18	3.52 ± 0.2	0.03	ns	0.8295	ns	**0.0009**	**

Asterisks indicate statistically significant differences between treatments * *p* < 0.05, ** *p* < 0.01; ns: no significant difference.
