# Supplementary figures and images for: JA Signaling Inhibitor JAZ Is Involved in Regulation of AM Symbiosis with Cassava, Including Symbiosis Establishment and Cassava Growth
Source: J Fungi (Basel). 2025 Aug 19;11(8):601. doi: 10.3390/jof11080601 (PMC12387459; doi:10.3390/jof11080601)

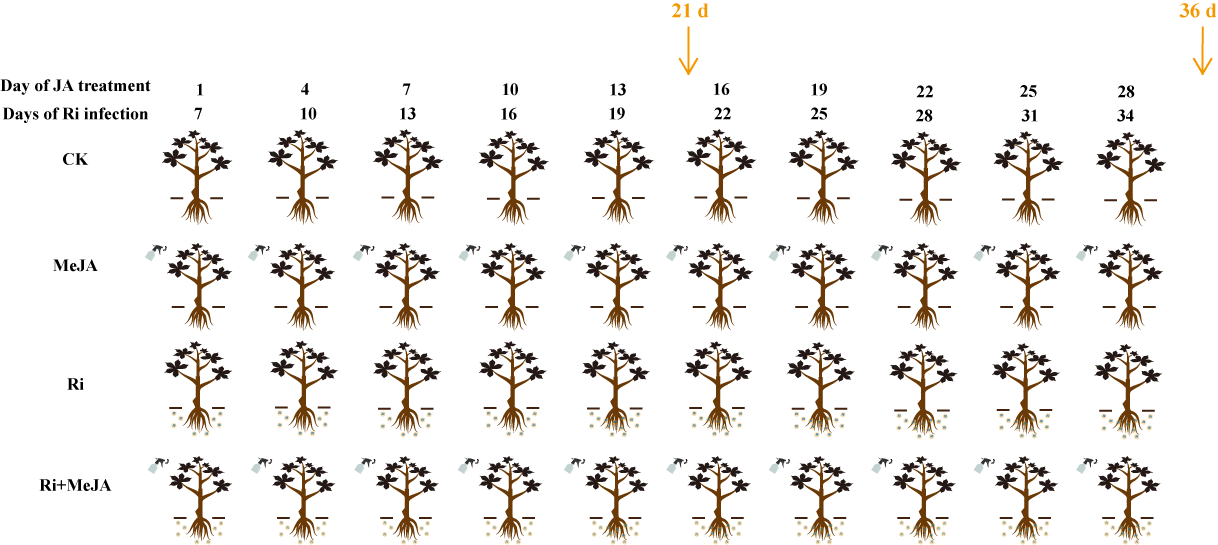

Supplement: Supplementary file 1 [file jof-11-00601-s001.zip › Figure S1.tif]

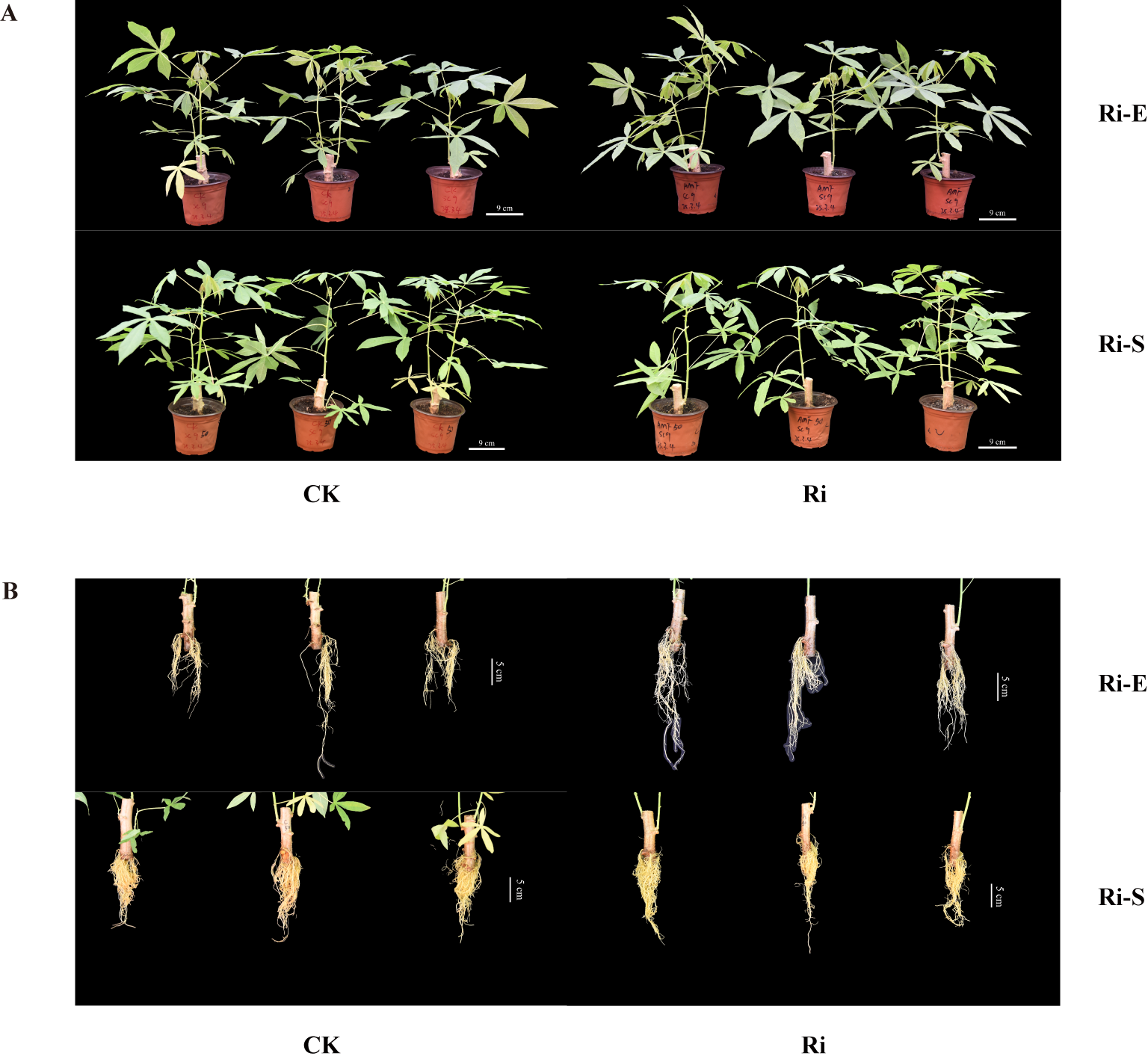

Supplement: Supplementary file 1 [file jof-11-00601-s001.zip › Figure S2.tif]

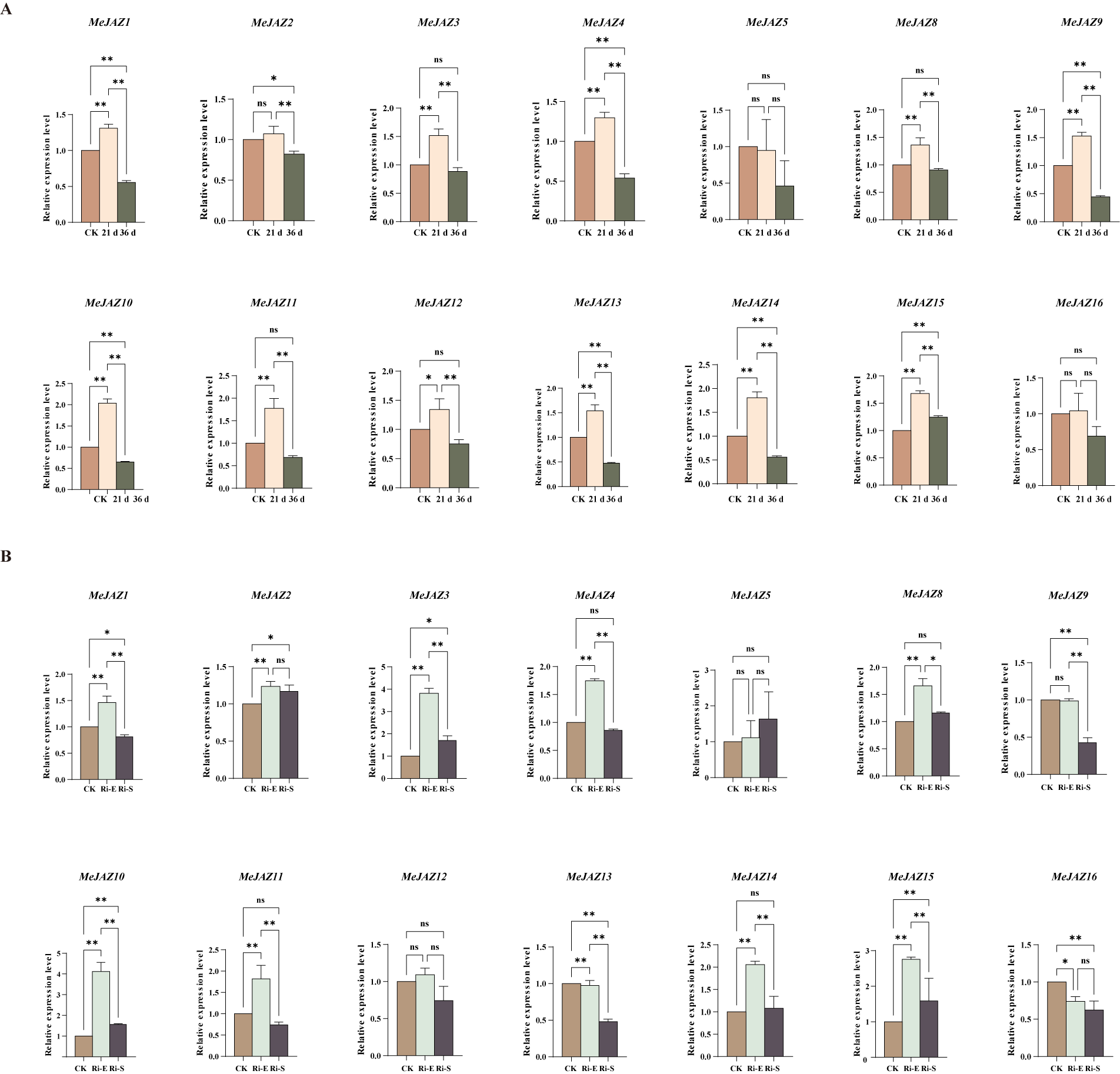

Supplement: Supplementary file 1 [file jof-11-00601-s001.zip › Figure S3.tif]

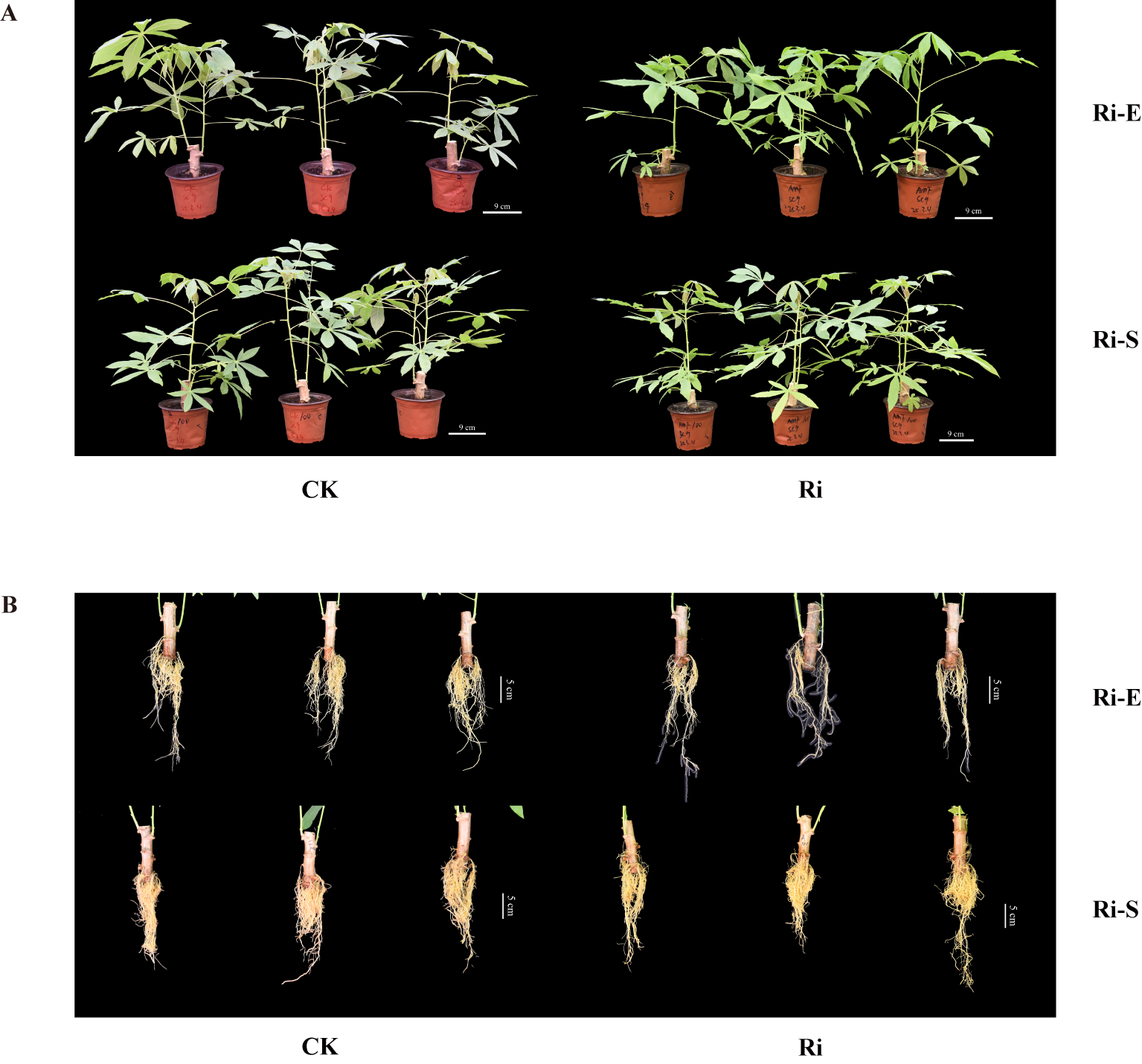

Supplement: Supplementary file 1 [file jof-11-00601-s001.zip › Figure S4.tif]

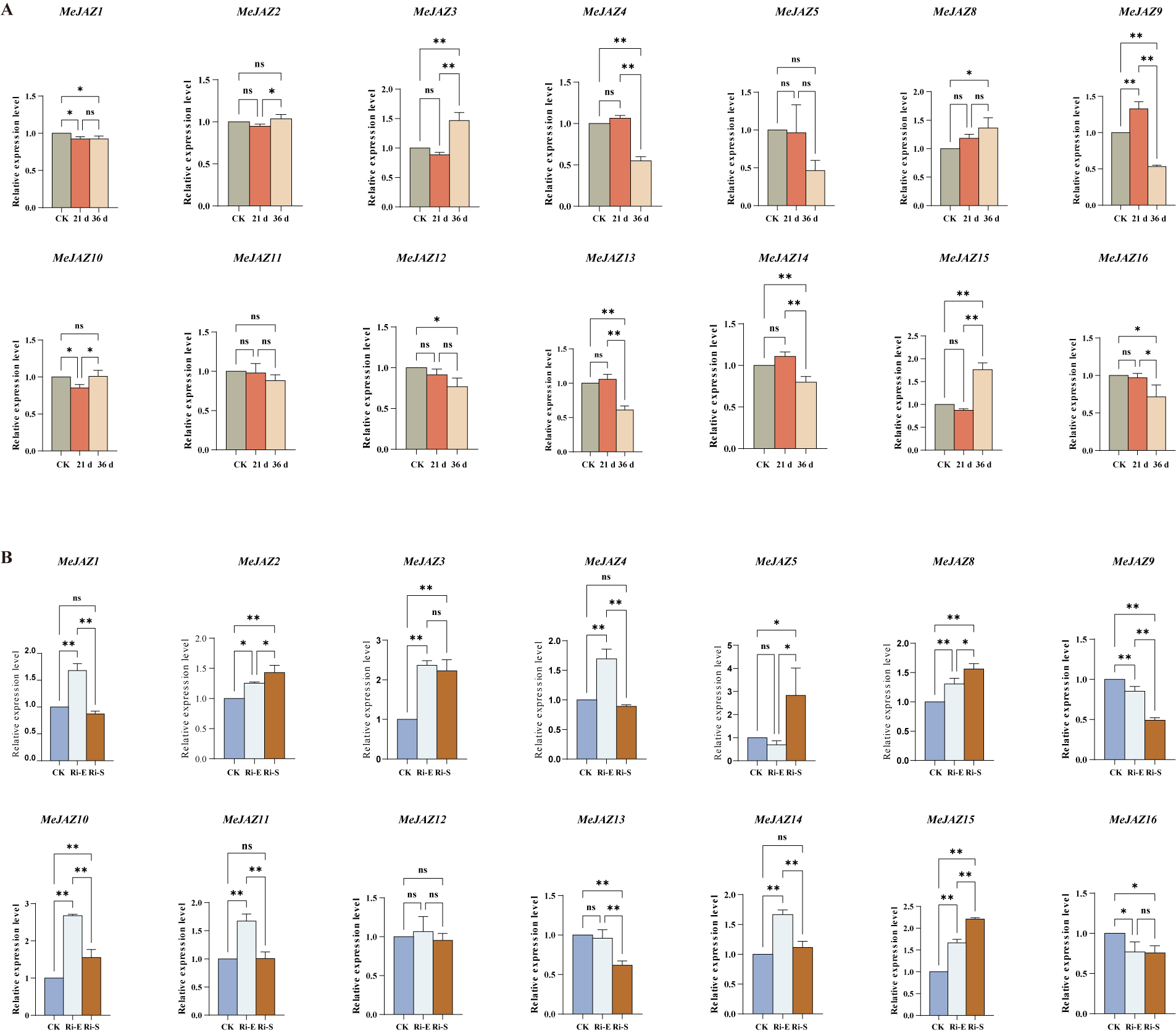

Supplement: Supplementary file 1 [file jof-11-00601-s001.zip › Figure S5.tif]

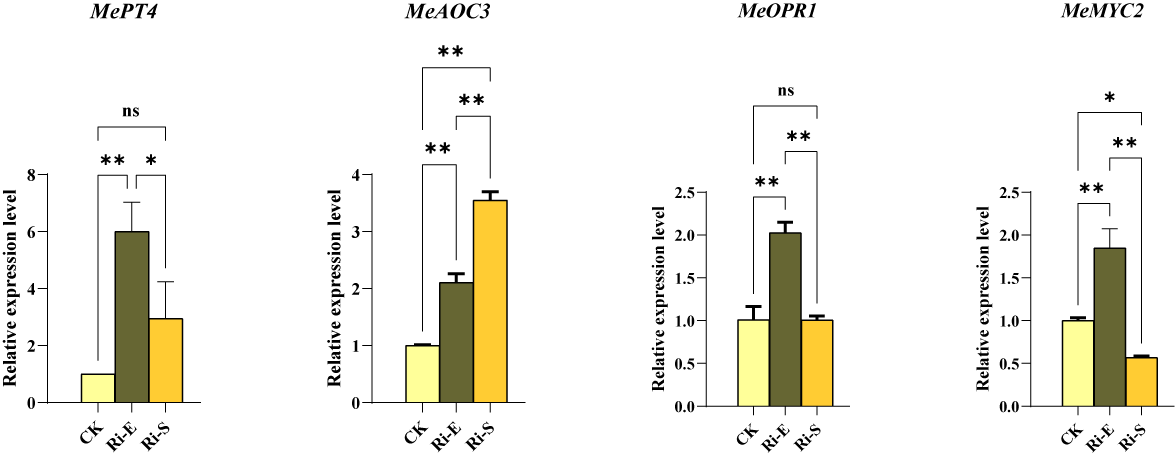

Supplement: Supplementary file 1 [file jof-11-00601-s001.zip › Figure S6.tif]
